# The Sweet Surrender: How Myeloid Cell Metabolic Plasticity Shapes the Tumor Microenvironment

**DOI:** 10.3389/fcell.2018.00168

**Published:** 2018-12-11

**Authors:** Je Lin Sieow, Sin Yee Gun, Siew Cheng Wong

**Affiliations:** ^1^Singapore Immunology Network, A^∗^STAR, Singapore, Singapore; ^2^School of Biological Sciences, Nanyang Technological University, Singapore, Singapore

**Keywords:** tumor immunology, macrophages, myeloid derived suppressor cell (MDSC), immunometabolism, immunotharapy, glycolysis

## Abstract

Immune cells are one of the most versatile cell types, as they can tailor their metabolic activity according to their required function. In response to diverse environmental cues, immune cells undergo metabolic reprogramming to support their differentiation, proliferation and pro-inflammatory effector functions. To meet a dramatic surge in energetic demand, immune cells rewire their metabolism to utilize aerobic glycolysis. This preferential use of glycolysis even under aerobic conditions is well established in tumor cells, and is known as the “Warburg effect.” Tumor cells avidly use glucose for aerobic glycolysis, thereby creating a nutrient-starved microenvironment, outcompeting T cells for glucose, and directly inhibiting T-cell anti-tumoral effector function. Given that both immune and tumor cells use similar modes of metabolism in the tumor stroma, it is imperative to identify a therapeutic window in which immune-cell and tumor-cell glycolysis can be specifically targeted. In this review, we focus on the Warburg metabolism as well as other metabolic pathways of myeloid cells, which comprise a notable niche in the tumor environment and promote the growth and metastasis of malignant tumors. We examine how differential immune-cell activation triggers metabolic fate, and detail how this forbidding microenvironment succeeds in shutting down the vigorous anti-tumoral response. Finally, we highlight emerging therapeutic concepts that aim to target immune-cell metabolism. Improving our understanding of immunometabolism and immune-cell commitment to specific metabolic fates will help identify alternative therapeutic approaches to battle this intractable disease.

## Immunometabolism: A Re-Emerging Field

Immunometabolism describes the interplay between immunologic and metabolic processes, and can be interpreted in two possible ways: (1) the role of metabolic changes in immune cells in influencing cellular functions and broader immunity (Buck et al., 2017), and (2) the role of immune cells in directing metabolism in organs or the whole organism (Pearce and Pearce, 2013). Here, we focus on the former and discuss how changes in the metabolic profile of immune cells can have an impact in cancer.

Immune cells respond to environmental cues (e.g., infection, tissue injury, cellular stress and tumor invasion) to assume a wide variety of functional states. Immune cells are a vital component of the body’s defense against disease and are important in maintaining tissue homeostasis. Upon encountering a stimulus, immune cells may migrate, proliferate, secrete cytokines and undergo apoptosis. Different immune-cell subsets utilize different metabolic pathways to generate the required cellular energy and biosynthetic macromolecules to fulfill their effector functions when mounting a host immune response.

Thus far, the metabolic pathways utilized by T cells have been the most well studied and recently reviewed (Bettonville et al., 2016; Bantug et al., 2018). As such, we focus primarily on myeloid cell metabolism, as these cells represent a substantial component of the innate immune system. Similar to cells of the adaptive immune system, myeloid cells undergo robust metabolic reprogramming upon activation and stimulation (Geeraerts et al., 2017; Stienstra et al., 2017). Myeloid cells are highly plastic, they can exhibit wide metabolic heterogeneity depending on the types of stimuli and microenvironment they encounter (Biswas, 2015). This diverse metabolic response gives rise to numerous phenotypes and polarization states that dictate downstream immune responses. Here, we highlight the metabolic pathways used by myeloid cells in the cancer setting, and discuss some of the current strategies to target myeloid-cell metabolism and improve the efficacy of cancer immunotherapies.

## Immune Cell Metabolic Pathways

Metabolic reprogramming is a hallmark of cancer (Hanahan and Weinberg, 2011). For tumor cells to thrive, they must reprogram their metabolic profiles to fuel their energy needs according to the microenvironment, and to promote their proliferation, survival and differentiation (Boroughs and DeBerardinis, 2015). One of the main metabolic pathways used by tumor cells is aerobic glycolysis, widely known as the Warburg effect (Sica et al., 2017). Likewise, immune cells can also utilize multiple metabolic pathways for energy production. For example, immune cells can undergo either glycolysis and/or oxidative phosphorylation (OXPHOS) to produce adenosine triphosphate (ATP) for their functional requirements. During glycolysis, cells uptake glucose in the environment via glucose transporters (GLUT), and convert it into pyruvate and ATP. To maintain glycolytic flux, cells convert pyruvate to lactate to regenerate nicotinamide adenine dinucleotide (NAD+). An intermediate molecule of glycolysis is glucose-6-phosphate (G6P) — the first molecule of the pentose phosphate pathway (PPP). The PPP consists of an oxidative and a non-oxidative branch (Patra and Hay, 2014). The oxidative branch generates reducing equivalents, such as nicotinamide adenine dinucleotide phosphate (NADPH) and ribose-5-phosphate (R5P) (Patra and Hay, 2014) while the non-oxidative branch coverts glycolytic intermediates, such as fructose-6-phosphate (F6P) and glyceraldehyde-3-phosphate (G3P) into pentose phosphates. The reversible nature of enzymes in the non-oxidative branch enables the PPP to utilize glycolysis according to the metabolic demands of a cell (Cho et al., 2018). By contrast, OXPHOS is an oxygen-driven process that produces 36 ATP per glucose molecule (O’Neill et al., 2016). OXPHOS takes place when energy precursors derived from acetyl-CoA generate and feed electrons into the electron transport chain (ETC), leading to phosphorylation of ADP to produce ATP.

Cells can also use glutamine or fatty acids as nutrients to fuel the TCA cycle (Geeraerts et al., 2017). For example, glutamine can be converted into α-ketoglutarate, an intermediate of the TCA cycle, while the fatty acid oxidation (FAO) pathway degrades fatty acids into acetyl-CoA for ATP production.

OXPHOS is a highly efficient pathway for ATP production, and is the preferred metabolic pathway utilized by cells with high energy demands (O’Neill et al., 2016). Conversely, glycolysis yields only two ATP per glucose molecule. Although glycolysis is not the most efficient way to produce energy, high glycolytic rates allow cells to produce sufficient energy and intermediates to fuel their growth and functional demands. Tumor cells are a typical cell-type that uses this approach, switching from OXPHOS to glycolysis even in the presence of oxygen. The Warburg effect enables tumor cells to adapt to their microenvironment for their survival and proliferation (Hanahan and Weinberg, 2011; Ward and Thompson, 2012).

Besides cellular energy and ATP production, intermediates produced from the different metabolic pathways, such as glycolysis, the PPP and the TCA cycle are also necessary for fatty acid and amino acid synthesis. For example the PPP and TCA cycles generate NADPH and citrate for fatty acid synthesis, respectively (O’Neill et al., 2016). The unique model of metabolism that each cell uses is dependent on their microenvironment and external stimuli.

Mechanistically, conceptual progress has been made in understanding the signaling pathways that underlie immune cell metabolism reprogramming. One such pathway is the mTOR pathway, which regulates various important cell processes such as protein synthesis, cell growth and metabolism (Saxton and Sabatini, 2017). Specifically, myelopoiesis requires mTOR signaling and loss of mTOR dampens innate immune responses against *Listeria monocytogenes* infection (Karmaus et al., 2017). Inhibition of the mTOR pathway with rapamycin in both human monocytes and dendritic cells prevented the anti-inflammatory effect and Th1 responses of glucocorticoids (Weichhart et al., 2011). The mTOR pathway is also a key orchestrator of myeloid cell effector responses to nutrient availability and cellular energy requirements, driving an increase in glucose utilization during glycolysis (Covarrubias et al., 2015). HIF-1α induces the over-expression of several glycolytic proteins including glucose transporters (i.e., GLUT1 and GLUT3), and enzymes such as hexokinase-1 (HK1), HK2 and LDHA in cancer cells (Marin-Hernandez et al., 2009). Likewise in macrophages, HIF1α enhances glycolytic pathway activity and lowers OXPHOS rate (Wang et al., 2017; Li et al., 2018). In cases where tumor growth exceeds the ability of the host’s vascular system to supply the tumor microenvironment with sufficient oxygen, hypoxic regions are established that induce HIF-1α activation and instruct cancer cells to utilize glucose causing an increase in lactate release (Eales et al., 2016).

## The Tumor Microenvironment and Myeloid Cells

**FIGURE 1:**
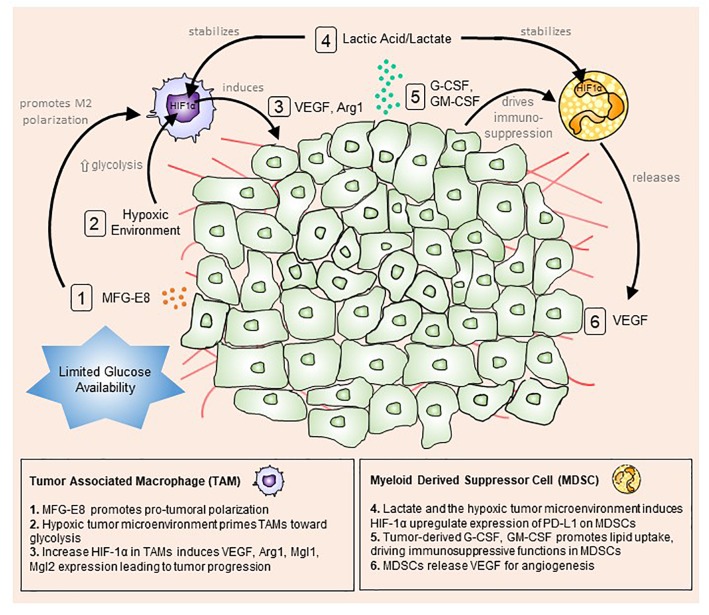
The tumor microenvironment primes myeloid cells toward a pro-tumoral phenotype. Tumor cells actively uptake surrounding glucose to drive aerobic glycolysis and fuel their growth and proliferation. This mode of metabolism creates a microenvironment with limited available glucose and oxygen. Stressed tumor cells undergo apoptosis and produce milk fat globule-EGF factor 8 protein (MFG-E8), which promotes alternative (M2) macrophage polarization (1). At the same time, hypoxic conditions trigger macrophages to up-regulate hypoxia-inducible factor 1-alpha (HIF-1α), promoting a glycolytic switch (2). Lactic acid/lactate, the by-product of glycolysis, stabilizes HIF1α in tumor-associated macrophages (TAMs) and myeloid-derived suppressor cells MDSCs (4). Increased HIF1α expression in TAMs enhances vascular endothelial growth factor (VEGF) and arginase 1 (Arg1) expression and secretion, which feedbacks to tumor cells to boost tumor progression (3). Conversely, tumor-derived granulocyte-colony stimulating factor (G-CSF) and granulocyte-macrophage colony-stimulating factor (GM-CSF) up-regulate lipid transport receptors to increase lipid metabolism and drive immunosuppressive functions in MDSCs (5). In turn, MDSCs release VEGF and cathepsin to induce angiogenesis and vasculogenesis (6).

The tumor microenvironment consists of a mix of tumor, immune and stromal cells, all of which contribute to shaping the pro-inflammatory state and promoting tumor initiation, progression and metastasis (Whiteside, 2008) (Figure 1).

### Macrophage Polarization in the Tumor Microenvironment

Macrophages are a prominent immune subset involved in many homeostatic and immune functions. These cells are highly plastic and thus can perform a wide diversity of functions (Wynn et al., 2013). Classical (M1) macrophages are activated primarily by IFN-γ and/or lipopolysaccharide (LPS), and produce pro-inflammatory cytokines, nitric oxide or reactive oxygen intermediates (ROI) to mount an immune response against bacteria and viruses. Alternative (M2) macrophages are activated by cytokines, such as interleukin (IL)-4 and IL-10. These macrophages are mainly associated with wound healing and tissue repair (Wynn et al., 2013).

Depending on the external stimuli, microenvironment and types of cytokine present, these myeloid cells can polarize into specialized subsets (Wynn et al., 2013). For example in prostate cancer, milk fat globule-EGF factor 8 (MFG-E8) secreted by tumor cells facilitates macrophage efferocytosis – a process of removing apoptotic cells and also promotes M2 polarization (Soki et al., 2014). Furthermore, the hypoxic microenvironment, created by highly glycolytic tumor cells, also triggers macrophage polarization toward an anti-inflammatory phenotype (Leblond et al., 2016). As such, strategies that can re-polarize these tumor-associated macrophages (TAMs) toward an anti-tumoral phenotype are advantageous for targeting tumor cells.

Using a murine model of pancreatic ductal adenocarcinoma (PDAC), Zhu and colleagues highlighted that TAMs are of heterogeneous origin (Zhu et al., 2017). They found that circulating blood monocyte-derived TAMs have a potent role in antigen presentation whereas tissue resident embryonically derived TAMs are preferentially involved in tissue repair and remodeling (Zhu et al., 2017). Recent studies observed that tumor-conditioned macrophages (TCMs) exhibit a mixed M1 and M2 phenotype (Penny et al., 2016), which underlies their capability to promote tumor progression and metastasis, whilst retaining their anti-tumoral function in the presence of tumor-targeting monoclonal antibodies (Grugan et al., 2012). Such tumor antigen-targeting antibody-dependent cellular phagocytosis is observed to be more superior in TCMs and M1 (IFNγ + LPS) macrophages as compared to M2 macrophages (IL-4 + IL-13) (Grugan et al., 2012). Besides tumor-targeting antibodies, a soluble SIRPα-Fc protein could also promote Fcγ receptor-mediated phagocytosis of cancer cells by macrophages (Lin G.H.Y. et al., 2017). Specifically, this soluble SIRPα-Fc triggers both M1 (IFNγ + LPS) and M2 (IL-10 + TGF-β) macrophages to significantly increase phagocytosis of lymphoma cells as compared to other macrophages populations (Lin G.H.Y. et al., 2017). A possible explanation for the difference in phagocytic nature of M2 (compared to M1) in both studies could be due to the use of unique M2 polarizing signals, i.e., IL-4 and IL-13 versus IL-10 and TGF-β. Again, these studies demonstrated that macrophages are plastic in nature and their phenotypes strongly dictate their functions. The diverse spectrum of differential TAM activation states in the tumor microenvironment highlights the potential of targeting and re-polarizing their pro-tumoral phenotype toward an anti-tumoral phenotype to augment tumor progression and metastasis.

### Metabolic Sensing of Macrophages Shapes Their Functional Phenotype

Numerous studies have suggested that the metabolic pathways used by macrophages regulate their immune function (Lampropoulou et al., 2016; Mills and O’Neill, 2016; Wang et al., 2017). Macrophages undergo metabolic adaptations to survive the harsh tumor microenvironment, resulting in differential phenotypes and downstream effector functions. M1 macrophages up-regulate glycolysis leading to the accumulation of succinate, while M2 macrophages up-regulate OXPHOS and FAO (Mills and O’Neill, 2016).

Metabolites generated during macrophage activation can impact and shape the inflammatory immune response. For instance, succinate, a metabolic product of the citric acid cycle accumulates in inflammatory macrophages to drive HIF-1α stabilization and IL-1β production (Tannahill et al., 2013). This metabolite, when secreted into the extracellular space, can further stimulate IL-1β secretion by macrophages in an autocrine and paracrine manner (Littlewood-Evans and Sarret, 2016). On the other hand, as a negative feedback mechanism, itaconate generated by LPS-stimulated macrophages suppresses inflammatory macrophages either by blocking succinate dehydrogenase-mediated oxidation (Lampropoulou et al., 2016) or by alkylation of the cysteine residue on repressor protein kelch-like ECH-associated protein 1 (KEAP1) to release transcription factor nuclear factor erythroid 2-related factor 2 (NRF2) from proteasomal degradation (Mills et al., 2018). This led to the eventual downregulated expression of HIF-1α and reduced production of pro-inflammatory proteins. Taken together, these extracellular metabolites can have a critical influence on macrophage activation and polarization. This phenomenon influences the way in which macrophages direct the immune response in the tumor microenvironment.

### Glycolysis and the Warburg Effect

Decades of work have focused on tumor-cell metabolism, but it was not until the last decade did we learn that the highly glycolytic nature of tumor cells results in a nutrient-limited microenvironment (Hanahan and Weinberg, 2011; Cantor and Sabatini, 2012; Ward and Thompson, 2012). Tumor-infiltrating macrophages are forced to compete with tumor cells particularly for glucose, and thus undergo reprogramming in their energy requirements and changes in their glucose metabolism (Netea-Maier et al., 2018). As such, macrophages alter their cellular bioenergetics to promote their re-polarization to a pro-inflammatory phenotype.

HIF-1α over-expression in macrophages upregulates glycolytic genes, such as pyruvate dehydrogenase kinase 1 (Pdk1), phosphoglycerate kinase 1 (Pgk1), glucose transporter 1 (Glut1), glucokinase (Gck) and pyruvate kinase isozymes M2 (Pkm2) (Wang et al., 2017). This resulted in an active glycolytic phenotype and decreases mitochondrial oxidation in these HIF-1α-overexpressing macrophages (Wang et al., 2017). LPS-activated monocytes reduce oxygen consumption rates and increase, to some degree, anaerobic glycolysis. However, as lactate concentration increases, LPS-induced glycolysis is abrogated in conjunction with down-regulation of pro-inflammatory markers, such as IL-1β, IL-6, IL-12β and CD40 in bone marrow-derived macrophages and peritoneal macrophages (Errea et al., 2016). In line with this, lactate derived from the PDAC cell line Panc-1, also promotes M2-like polarization of THP-1 derived macrophages (Ye et al., 2018). In addition, blocking glycolysis with 2-deoxyglucose (2DG) — a competitive inhibitor of hexokinase 2 (HK2) that catalyzes the rate-limiting step of glycolysis — disrupts glycolytic flux in activated monocytes and impedes TNFα secretion (Dietl et al., 2010). Our group previously showed that PDAC tumor-conditioned macrophages, differentiated from CD14+ human peripheral blood monocytes, exhibit a pro-metastatic phenotype (Penny et al., 2016). In contrast to macrophages differentiated in media of human pancreatic normal epithelia, PDAC tumor-conditioned macrophages promoted angiogenesis, enhanced epithelial-to-mesenchymal transition and increased the extravasation of PDAC cells out of blood vessels (Penny et al., 2016). Interestingly, PDAC-differentiated macrophages also showed an increased glycolytic capacity, and 2DG treatment abrogated the pro-tumoral function of these cells (Penny et al., 2016). These findings suggest that changes in the metabolic profiles of monocytes and macrophages have an important role in the functional output of these cells toward a pro-metastatic phenotype.

During aerobic glycolysis (the Warburg effect), pyruvate is converted to lactic acid by lactate dehydrogenase A (LDHA). Lactic acid, an end product of both aerobic and anaerobic glycolysis, activates vascular endothelial growth factor (VEGF), transforming growth factor β (TGF-β), and HIF-1α in oxidative tumor cells (Dietl et al., 2010). At the same time, lactic acid released by glycolytic cancer cells into the tumor microenvironment also stabilize HIF-1α expression in bone-marrow derived macrophages (Dietl et al., 2010). Colegio and colleagues further demonstrated that HIF-1α expression in tumor macrophages induce M2-associated genes such as VEGF, arginase 1 (Arg1), macrophage galactose-type lectin-1 (Mgl1) and macrophage galactose-type lectin-2 (Mgl2) in a colon carcinoma mouse model. Human breast cancer cell line-derived lactate also up-regulate the expression of the M2 markers CD163 and CD206, and down-regulate the M1 marker HLA-DRα in human THP-1 monocytic cells (Lin S. et al., 2017). In the same study, they also reported that chemokine CCL5 is up-regulated, and this specific CCL5–CCR5 axis can govern cancer cell metastasis *in vivo* and *in vitro* (Lin S. et al., 2017). These data highlights the pivotal role that metabolic derivatives have in remodeling the immune system, by acting on the differentiation, recruitment, activation and polarization of tumor-infiltrating macrophages.

A study by Wenes et al. (2016) showed that nutrients abundance (such as glucose) within the tumor microenvironment could affect blood vessels formation. Using *in vivo* mouse models for subcutaneous Lewis Lung carcinoma, orthotopic breast cancer and spontaneous mammary tumor, they observed that REDD1, an inhibitor of mTORC1 is highly upregulated in TAMs, particularly those located in hypoxic regions. Depletion of REDD1 in these macrophages greatly enhanced their glycolysis and they competed with neighboring endothelial cells for glucose. The restriction in glucose availability for the endothelial cells, in turn, stabilizes the vascular network to inhibit metastasis. These findings are in contrast to those reported by our group, where inhibiting glycolysis in TAMs resulted in a decrease in their pro-metastatic phenotype (Penny et al., 2016). At first glance, it appears that Wenes and colleagues drew a contradictory conclusion to that of our group; however, many experimental differences may account for this inconsistency. In our *in vitro* study, peripheral blood monocytes from healthy donors were stimulated with tumor-conditioned media under normoxic conditions to derive TCMs (Penny et al., 2016). On the other hand, Wenes et al. (2016) utilized an *in vitro* system where bone-marrow derived macrophages were stimulated with conditioned media and incubated in either normoxic or hypoxic culture conditions. Another major difference in these two studies is the glycolytic state of the TCMs: the study by Penny et al. (2016) was blocking macrophage glycolysis while the REDD1-deficient macrophages in the study by Wenes et al. (2016) further enhances their glycolysis. Despite contradictory results, these studies nevertheless provide some insight as to how metabolic rewiring in TAMs can affect tumor metastasis and progression. As such, current studies and strategies are targeted toward the glycolysis of macrophages to skew TAMs away from a pro-tumoral toward an anti-tumoral phenotype to impart a positive impact on tumor outcome.

### Amino Acid Metabolism

Targeting amino acid metabolism in cancer cells has the potential to confer metabolic control and regulation of the tumor microenvironment. Amino acid metabolic enzymes are regulated by tumor suppressors and oncogenes, and have thus been exploited as targets for cancer treatment (Ananieva, 2015). The different macrophage polarized states exhibit readily distinguishable modes of L-arginine (L-Arg) metabolism, giving rise to differential macrophage functions (Rath et al., 2014). For example, IFNγ and LPS-stimulated macrophages up-regulate inducible nitric oxide synthase (iNOS), which catalyzes the conversion of L-Arg into nitric oxide (NO) and L-citrulline. On the other hand, lL-4-polarized macrophages up-regulate Arg1, which catalyzes the conversion of L-Arg to L-ornithine, and polyamine synthesis (Modolell et al., 1995). NO release by macrophages contributes to TAM anti-tumoral activity, whereas polyamines promote tumor-cell growth and progression (Chang et al., 2001). Macrophage Arg1 expression also enhances tumor-cell growth and suppresses tumor cytotoxicity by inhibiting NO production (Chang et al., 2001). Indeed, Arg1 expression by TAMs mediates T-cell immunosuppression (Kusmartsev and Gabrilovich, 2005). Taken together, data suggest that L-Arg metabolism in macrophages can either enhance tumor cell growth by providing tumors with polyamines or suppress cytotoxicity of tumor cells by reducing NO production. Depending on the macrophage polarization state in the tumor microenvironment, amino acid metabolism can have differential effects on tumor progression.

### Lipid Metabolism

Tumor-associated fatty acid synthase (FASN) is a key lipogenic enzyme that catalyzes the terminal steps of fatty acid biogenesis and confers a growth and survival advantage to cancer cells (Menendez and Lupu, 2007). Cancer cells adapt and undergo changes in their lipid metabolism; they acquire a lipogenic phenotype by expressing high levels of monoacylglycerol lipase (MAGL), which regulates the pro-tumorigenic lipid network that supports tumor progression (Nomura et al., 2010). Similar to tumor cells, macrophages also alter their lipid metabolism in response to microenvironmental stimuli (Dennis et al., 2010). For example, IL-4-activated macrophages, but not IFNγ-activated or LPS-activated mouse macrophages up-regulate fatty acid uptake and FAO (Odegaard and Chawla, 2011). In the context of the tumor microenvironment, FASN expression by TAMs polarized cells toward a pro-tumoral phenotype expressing IL-10 (Park et al., 2015). FASN was also shown to be an upstream regulator of peroxisome proliferator-activated receptor gamma (PPAR)-β/δ in myeloid cells, and myeloid cell-specific PPAR-β/δ knockout reduced tumor burden (Park et al., 2015). Other reports have shown that myeloid cells in tumor-bearing hosts possess high levels of triglycerides and cytoplasmic lipid droplets compared to cells from tumor-free mice and healthy individuals (Herber et al., 2010). These findings suggest that by altering lipid metabolism and the lipid levels in professional antigen-presenting cells, the functional activity and anti-tumoral immune response can be restored. Targeting TAM metabolism in tumor growth may thus be an important molecular mechanism in directing their anti-tumoral activity.

## Characteristics of Myeloid-Derived Suppressor Cells in Tumor Sites

The role that myeloid-derived suppressor cells (MDSCs) have in regulating tumor progression is well recognized (Gabrilovich et al., 2012; Ugel et al., 2015). MDSCs represent a heterogeneous population of early myeloid progenitors, immature granulocytes, macrophages and dendritic cells at different stages of differentiation that are distinct from mature myeloid cells, and can be functionally described by their strong immunosuppressive properties (Gabrilovich et al., 2012). MDSCs strongly expand as a result of perturbed hematopoiesis in pathological diseases, such as chronic inflammation and cancer, and were originally characterized in tumor-bearing mice as having a CD11b+Gr1+ phenotype (Ugel et al., 2015). MDSCs accumulate in the blood, bone marrow, and the peripheral lymphoid organs (including the lymph nodes and the spleen of tumor-bearing mice), where they have a causative role in promoting immune suppression and thus tumor progression (Ugel et al., 2015). MDSCs may potentially serve as a cellular target to control tumor cell growth.

MDSCs are comprised of two major subsets: monocytic MDSCs (M-MDSCs) and granulocytic MDSC (G-MDSCs). Both subsets can suppress the cytotoxic activity of cytotoxic CD8+ T lymphocytes (CTLs) and natural killer (NK) cells (Gabrilovich et al., 2012). In mice, M-MDSCs are classified as CD11b+Ly6ChighLy6G- cells, while G-MDSCs are classified as CD11b+Ly6ClowLy6G+ cells. In humans, M-MDSCs are CD33+CD14+CD15-HLA-DRlow while G-MDSCs are CD33+CD14-CD15+CD66b+HLA-DR-/low (Bronte et al., 2016). Despite extensive research on these immune cells, the cellular definition of MDSCs subsets remains to be elucidated. At the morphological level, M-MDSCs and G-MDSCs are identical to monocytes and granulocytes, respectively and there are no specific markers that can unequivocally differentiate them. Consequently, researchers are reluctant to use the current MDSC nomenclature to identify myeloid cells with immuno-suppressive capabilities (Coffelt et al., 2016; Porta et al., 2018).

Over the course of inflammation, neutrophils engage in various cell–cell interactions with other immune cells, such as macrophages, dendritic cells and lymphocytes (Mantovani et al., 2011). Neutrophils are innate immune cells involved in the first line of defense at the site of infection, and account for up to 60% of all leukocytes in the circulation. Neutrophils were traditionally seen as terminally differentiated effector cells that have a major role in microbial immunity and acute inflammation (Rosales, 2018). However, it now seems that these short-lived cells can function as immunosuppressive cells in the chronic, progressive disease such as cancer (Nagaraj et al., 2010; Rosales, 2018). Neutrophils secrete cytokines and myeloperoxidase, which is involved in monocyte and macrophage recruitment (Kolarova et al., 2013). Various studies have shown that tumor associated neutrophils (TANs) and their myeloid precursors, G-MDSCs, have important roles in tumor progression (Gregory and Houghton, 2011; Fridlender et al., 2012).

The tumor microenvironment polarizes TAMs toward a pro-tumoral M2-like phenotype, capable of promoting epithelial-to-mesenchymal transition in early pancreatic initiation and development (Helm et al., 2014a). Similar to macrophages, neutrophil plasticity has also been reported with data supporting a skewing of neutrophil phenotypes (Fridlender et al., 2009). TANs acquire a pro-tumoral phenotype to become N2-like neutrophils. These N2-like neutrophils favor tumor initiation and progression by releasing VEGF for angiogenesis, and expressing arginase to suppress cytotoxic T-cell activity (Galdiero et al., 2013). This polarization is largely dependent on TGF-β: during TGF-β blockade, neutrophils acquire an anti-tumor phenotype to become N1-like TANs (Fridlender et al., 2009). Anti-tumor N1-like TANs produce elevated amounts of tumor necrosis factor alpha (TNF-α), macrophage inflammatory proteins-1 alpha (MIP-1α), hydrogen peroxide and NO that are cytotoxic to tumor cells (Jablonska et al., 2010).

In a recent study, Sagiv et al. (2015) distinguished circulating murine neutrophils according to their density. The researchers characterized high-density neutrophils (HDNs) as N1-like cells and circulating low-density neutrophils (LDNs) as N2-like pro-tumoral cells. LDNs can be further subdivided into mature and immature cells, with the immature cells being referred to as G-MDSCs previously (Sagiv et al., 2015). Taken together, these data implicate neutrophil plasticity in mediating cancer. There is now major interest in understanding and characterizing neutrophil infiltration and polarization in cancer progression.

## Metabolic Alterations of Tumor-Infiltrating MDSCs

The high concentration of lactate produced when tumor cells undergo Warburg metabolism as well as the hypoxic tumor microenvironment promote tumor progression by modifying the anti-tumoral immune response through priming tumor-infiltrating neutrophils toward an immunosuppressive state resulting in the induction of MDSCs (Husain et al., 2013b). In turn, MDSCs inhibit CTLs and NK cell activities (Husain et al., 2013a; Umansky et al., 2016). MDSCs promote immune system dysfunction either by (1) depriving T cells of essential metabolites, such as arginine, tryptophan, and cysteine; (2) interfering with T-cell migration and stimulation; or (3) activating other pro-tumoral immune cells, such as regulatory T cells or TAMs (Rodriguez et al., 2017).

A study conducted by Corzo and colleagues showed that tumor-infiltrating MDSCs suppressed both antigen-specific and non-specific T-cell activity, which was accompanied by up-regulation of Arg1 and iNOS, and down-regulation of NADPH oxidase and reactive oxygen species (ROS) (Corzo et al., 2010). The mechanism for regulating the function and differentiation of MDSCs in the tumor microenvironment involves the transcriptional factor HIF-1α (Corzo et al., 2010). Moreover, lactate-induced HIF-1α contributes to suppressing adaptive immunity by promoting immuno-suppression via inducing expression of programmed death-ligand 1 (PD-L1) on MDSCs (Noman et al., 2014). The hypoxic tumor microenvironment causes selective up-regulation of PD-L1 on splenic MDSCs and PD-L1 blockade could enhance MDSC-mediated T-cell activation, accompanied by the concomitant down-regulation of immuno-suppressive cytokines IL-6 and IL-10 secreted by MDSCs.

Another key sensors of cellular energy metabolism is adenosine monophosphate-activated protein kinase (AMPK) (Long and Zierath, 2006). AMPK is activated by various stimuli, such as hypoxia and oxidative stress. In the context of MDSCs, murine cells exposed to OSU-53, a PPAR-inactive derivative that stimulates AMPK kinase modulated their function (Trikha et al., 2016). Specifically, increased AMPK phosphorylation reduced NO production, inhibited MDSC migration, and reduced IL-6 levels. This role for AMPK in regulating murine MDSC effector functions by dampening their immunosuppressive functions hence promoting T-cell proliferation may represent a novel role for AMPK in metabolic reprogramming of MDSCs (Trikha et al., 2016).

### Glycolysis and the Warburg Effect

Tumor cells derived lactic acid (the final product of glycolysis) mediated by HIF-1α can acts as an immunosuppressive metabolite and direct differential myeloid cell functions such as M2-like polarization (Colegio et al., 2014). Neutrophils rely almost exclusively on glycolysis and are strongly committed to anaerobic glycolysis for energy production (Pelletier et al., 2014) and effector functions, such as respiratory burst and chemotaxis (Jun et al., 2014). Neutrophils exhibit very low rates of OXPHOS due to the presence of only a few mitochondria per cell (Borregaard and Herlin, 1982). A study conducted by Azevedo et al. (2015) showed how PPP and glycolysis contribute to the formation of neutrophil extracellular traps (NETs). They demonstrated that a metabolic shift toward PPP is essential, as glucose-6-phosphate dehydrogenase (G6PD) release will fuel NADPH oxidase activity to ultimately produce superoxide (SO) that induces NET formation (Azevedo et al., 2015).

Both M-MDSCs and G-MDSCs in tumor-bearing mice exhibit higher rates of glycolysis compared to their normal mature cell counterparts in healthy mice. By up-regulating glycolytic genes in response to tumor-derived factors and down-regulating ROS production to protect from apoptosis, MDSCs can accumulate in tumors (Jian et al., 2017). Phosphoenolpyruvate, a glycolytic metabolite, is also involved in MDSC proliferation and survival status (Jian et al., 2017).

The glucocorticoid receptor (GR) is expressed by almost every cellular organism and is involved in regulating the genes that control energy metabolism and the immune response (Liao et al., 2014; Lu et al., 2017). GR signaling suppresses HIF-1α and regulates MDSC function via HIF-1α-dependent glycolysis, thus revealing a role for GR-HIF-1α axis in the metabolism and suppressive activities of MDSCs (Lu et al., 2017). These studies indicate that the regulation of glycolysis and its metabolites are able to direct downstream MDSC effector functions.

### Amino Acid Metabolism

The depletion of arginine through Arg1 was the first T-cell suppressive mechanism described in G-MDSCs, as these cells are the major source of Arg1 (Rodriguez et al., 2004, 2009). MDSC activation is initiated in response to IFNγ produced by anti-tumoral T cells in the tumor microenvironment (Wu et al., 2012). Once activated, MDSCs uptake large amounts of l-Arg by inducing the cationic amino acid transporter 2 (Cat2), Arg1 and iNOS (Cimen Bozkus et al., 2015). Rodriguez et al. (2004) showed that L-Arg depletion by G-MDSCs blocks CD3zeta expression in T cells resulting in the inhibition of antigen-specific proliferation (Rodriguez et al., 2004). As such, Arg1 production by G-MDSCs in the tumor microenvironment may represent a target for tumor evasion. Besides targeting Arg1, Cat2 ablation was shown to block L-Arg uptake, and as a result, impairs MDSC immunosuppressive and pro-tumoral activities (Cimen Bozkus et al., 2015).

Myeloid-derived suppressor cells can also sequester L-cysteine, causing its deprivation in the microenvironment (Srivastava et al., 2010). L-cysteine deprivation decreases the expression of CD3zeta and inhibits T-cell proliferation (Srivastava et al., 2010). As a result, MDSCs can effectively block T-cell activation by sequestering cysteine, as T cells lack the cystathionase required to convert methionine to cysteine.

The mTOR-signaling pathway can sense a decrease in amino acid metabolism and metabolites making it an important checkpoint that governs cell-cycle entry (Chantranupong et al., 2016). In the context of a tumor, restraining mTOR-mediated T-cell responses by depleting amino acids in the microenvironment may prevent the anti-tumoral immune response (Chantranupong et al., 2016). Besides mTOR, general control non-derepressible 2 (GCN2) kinase expressed by T cells, detects and responds to the immuno-regulatory signals generated by indoleamine 2,3-dioxygenase (IDO), and inhibits T-cell proliferation (Munn et al., 2005). MDSCs express high levels of IDO in response to IFNγ production and activation in the tumor microenvironment, creating yet another mechanism that drives their immunosuppressive phenotype during tumor progression (Pinton et al., 2016).

The multifaceted regulation and suppression of anti-tumoral T-cell responses by MDSC L-Arg metabolism provides several potential areas for therapeutic intervention. One example is the enzymatic depletion of L-Arg in cancer patients via the administration of a pegylated form of the catabolic enzyme arginase I (peg-Arg I), which has shown some therapeutic potential (Hernandez et al., 2010). Metabolites derived from L-Arg metabolism, such as cysteine and tryptophan, are also important regulators of MDSC immunosuppressive activity (Wu et al., 2012). Several studies have also revealed crucial roles for arginine, serine and glycine in driving T-cell proliferation and anti-tumoral activity (Stachlewitz et al., 2000; Geiger et al., 2016; Ma et al., 2017), however, their roles in mediating MDSC immunosuppressive activity remains to be explored.

### Lipid Metabolism

Another important metabolic pathway utilized by tumor cells is lipid metabolism, where lipid β-oxidation is one of the most efficient ways to generate ATP and fuel the cellular energy needs of tumor cells (Nieman et al., 2011; Li and Kang, 2017). Lipids and lipoprotein metabolites in the tumor microenvironment are important mediators of immune-cell function. For example, tumor-resident dendritic cells accumulate oxidized lipoproteins via scavenger receptor-mediated internalization and form lipid droplets (Ramakrishnan et al., 2014; Cubillos-Ruiz et al., 2015). Also, expression of lectin-like oxidized low-density lipoprotein receptor-1 (LOX-1) by MDSCs enable these cells to specifically associate with endoplasmic reticulum (ER) stress and lipid metabolism, which posses potent immuno suppressive activity promoting T-cell suppressive functions (Condamine et al., 2016). More recently, it has been shown in MDSCs that PPAR-γ has an important role in neutral lipid metabolism signaling controlled by lysosomal acid lipase (Zhao et al., 2016). Zhao et al. (2016) showed that enhanced PPAR-γ activity restrains ROS production by G-MDSCs, thereby impairing cancer cell proliferation and metastasis. In addition, increased exogenous lipid uptake and FAO causes tumor-infiltrating MDSCs to undergo both metabolic and functional reprogramming to become highly immunosuppressive cells (Al-Khami et al., 2017). Tumor-derived granulocyte-colony stimulating factor (G-CSF) and granulocyte-macrophage colony-stimulating factor (GM-CSF) together with signal transducer and activator of transcription 3 and 5 (STAT3 and STAT5) signaling induces lipid transport receptor up-regulation, which increases lipid uptake in the tumor microenvironment (Al-Khami et al., 2017). Inhibiting STAT3 or STAT5 signaling or knockout of CD36, a fatty acid translocase, can prevent lipid metabolism and thus the immunosuppressive functions of MDSCs in the tumor environment. This effect results in a CTL-dependent reduction in tumor kinetics.

Taken together, tumor-derived metabolites and fatty acid derivatives in the tumor environment can reprogram and cause downstream functional changes in MDSCs. As fatty acids are largely involved in tumor progression and survival, including important roles in providing the necessary energy and macromolecules for membrane synthesis (Liu et al., 2017), approaches that target lipid metabolism may have promise in rewiring the pro-tumoral phenotype of MDSCs toward a more favorable anti-tumoral one.

In summary, we consider that understanding the immunometabolism of MDSCs is of pivotal and crucial importance, as targeting their metabolic profiles will widen the therapeutic armamentarium for cancer patients. Reprogramming the downstream suppressive effector functions of MDSCs within the tumor microenvironment may be a novel target for tumor resistance.

## Myeloid Cells Influence Anti-Tumoral Immunity

**FIGURE 2:**
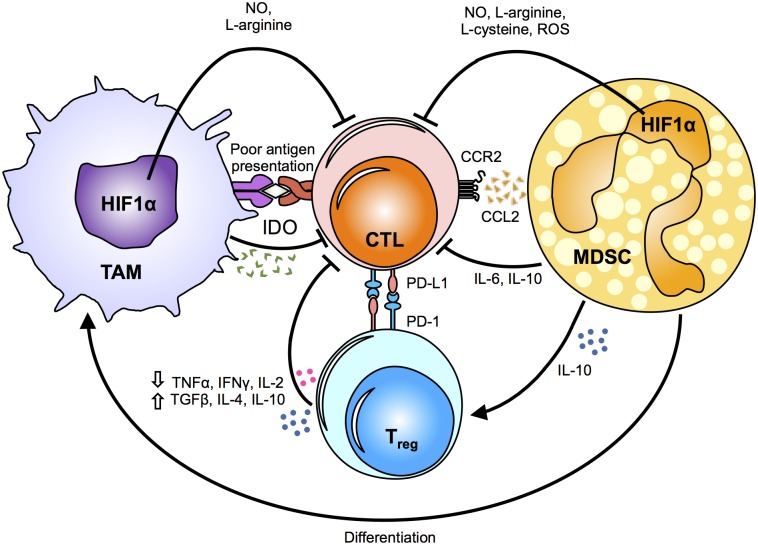
Mechanisms of myeloid cell suppression in the tumor. The tumor microenvironment primes myeloid cells, such as tumor-associated macrophages (TAMs) and myeloid derived suppressor cells (MDSCs), by driving their function towards a pro-tumoral phenotype. The metabolic fates and alterations in myeloid cells caused by changes in the hypoxic tumor microenvironment induce the release of metabolic intermediates, such as nitric oxide (NO), L-arginine, L-cysteine and reactive oxygen species (ROS), which in turn modulate cytotoxic T-lymphocyte (CTL) effector responses. Notably, indoleamine 2,3-dioxygenase (IDO) secretion suppresses antigen-specific T-cell expansion. Chemokines and cytokines secreted by TAMs and MSDCs directly and/or indirectly inhibit the anti-tumoral cytotoxic responses of CTLs by inducing regulatory T cells (T_regs_). In addition, PD-1 up-regulation on T_regs_ renders them as highly immunosuppressive cells. The critical role of TAMs and MDSCs in suppressing the anti-tumoral responses of CTLs supports that changes in their metabolic profile can influence the release of various cytokines and metabolic intermediates, and ultimately affect tumor growth, metastasis and drug resistance. HIF1α, hypoxia-inducible factor 1-alpha; TNFα, tumor necrosis factor alpha; IFNγ, Interferon gamma; IL, interleukin; TGFβ, transforming growth factor beta; CCR2, C-C chemokine receptor type 2; CCL2, chemokine (C-C motif) ligand 2; PD-L1, programmed death-ligand 1; PD-1, programmed cell death protein 1.

Anti-tumoral immune responses are mainly directed by cytotoxic CD8+ T cells and NK cells. However, the tumor microenvironment is often unsupportive of these cancer-killing cells, with TAMs and MDSCs having a critical role in suppressing their cytolytic functions (Figure 2).

### TAMs on T Cells

Tumor-associated macrophages repress anti-tumoral cellular activity mainly through (1) suppressing polyclonal T-cell proliferation and (2) promoting an immunosuppressive T-cell phenotype in the tumor. Macrophages are the most abundant population in the tumor microenvironment, and have a crucial role in regulating the T-cell response. Unfortunately, both M1-like (MHCII^high^) and M2-like (MHCII^low^) TAMs are poor antigen presenters and are ineffective at stimulating naïve T-cell proliferation (Movahedi et al., 2010). In the hypoxic tumor stroma, up-regulated HIF-1α and STAT1 expression in TAMs (Doedens et al., 2010) triggers NO secretion (Kusmartsev and Gabrilovich, 2005; Movahedi et al., 2010) and induces arginase activity respectively to induce T-cell apoptosis (Kusmartsev and Gabrilovich, 2005) and halt their expansion. Even if successful activation occurs, proinflammatory CD69+ T cells were found to promote tumor progression. Crosstalk between CD69+ T cells and TAMs up-regulates IL-12, IFNγ and TNF-α, which collectively potentiate IDO expression in TAMs to suppress antigen-specific T-cell expansion (Zhao et al., 2012).

Tumor-associated macrophages also help tumor cells evade immuno-surveillance by suppressing T-cell activity. Regulatory T cells (T_reg_) are immunosuppressive cells that are critical for maintaining self-tolerance, especially in autoimmune diseases. In the context of cancer, the presence of T_reg_ is often a negative prognostic factor with respect to overall survival (Waniczek et al., 2017). Su and colleagues demonstrated that T_reg_ develop from naïve CD4+ T cells *in situ*. More importantly, their study using a human breast cancer xenograft mouse model showed that naïve T-cell recruitment is directed by CCL18 produced by TAMs (Su et al., 2017). It has also been reported that CD163^high^ TAMs induce blood-derived CD4+ T cells to secrete less IL-2 and more TGF-β, IL-10 and IL-4 (Dannenmann et al., 2013). Besides promoting T_reg_ function, CD163+ TAMs (M1-like) located around tumor/connective tissue also strongly secrete IL-10 and express more PD-L1 compared to other TAM subsets found within the tumor (Kubota and Moriyama, 2017). Collectively, TAMs hijack T-cell physiological regulatory mechanisms to create a pro-tumoral microenvironment that promotes tumor progression.

The other arm of host anti-tumor immune response in human cancer is mediated by NK cells. A previous study by Mattiola and colleagues highlighted the effect of different macrophage subsets on NK-cell activity (Mattiola et al., 2015). They found that M1 macrophages specifically promoted IL-23 and IFN-β-dependent up-regulation of NKG2D, IL-1β-dependent up-regulation of NKp44, and trans-presentation of IL-15. These synergistic cellular mechanisms lead to enhance NK cell cytotoxicity (Mattiola et al., 2015). Conversely, co-culture of TAMs and NK cells stimulate macrophages to produce immunosuppressive TGF-β which induced exhaustion of NK cells characterized by decreased IFNγ, TNFα, and Ki-67 expression, and an overall reduction in NK-cell activation and cytotoxicity (Krneta et al., 2017; Peng et al., 2017). Evidently, contact between TAMs and NK cells downplays their cytotoxic function and promotes a skew toward a pro-tumoral response.

### MDSCs on T Cells

As discussed, MDSCs exert immune suppression via multiple mechanisms, such as NO production (Li et al., 2015), L-Arg depletion (Abdelaal et al., 2017) and L-cysteine sequestration (Srivastava et al., 2010). It seems that MDSCs essentially limit nutrient availability and deprive T cells of the energy source required to support their function. Interestingly, reactive nitrogen species produced by MDSCs induce post-translational modifications in CCL2 that decrease its affinity to CCR2 and its chemoattractant effects on CD8+ T cells, but not myeloid cells, into the tumor (Molon et al., 2011). Even after overcoming the hurdle of tumor infiltration, cell–cell contact between MDSCs and tumor-infiltrating lymphocytes inhibits effector-cell differentiation that is independent of activation status, IL-2 production or T-cell receptor signaling (Raber et al., 2016). Moreover, the hypoxic tumor stroma stabilizes HIF-1α in MDSCs promoting their rapid cellular differentiation into TAMs (Corzo et al., 2010). As such, intratumoral T cells not only endure nutrient deprivation, but are surrounded by immunosuppressive MDSCs and TAMs that douse them with suppressive cytokines. IL-10 secretion by immunosuppressed T cells up-regulates PD-L1 expression on MDSCs, in turn leading to T-cell dysfunction (Pinton et al., 2016).

## Tumor Associated Myeloid Cell Undermines Efficacy of Chemotherapy

### TAMs in Chemotherapy

Conventional cancer modalities, such as radiotherapy and chemotherapy, focus on killing dividing tumor cells. As scientists gather more information on cancer immunology, it has become apparent that TAMs are chemoprotective against these cancer-cell-centered treatments. Untargeted anti-neoplastic treatment not only eradicates cancer cells, but also impact surrounding normal tissue and immune cells. As discussed, macrophages are the most abundant immune cell populating the tumor tissue; they are exceptionally plastic cells that can differentiate into either pro-inflammatory or suppressive phenotypes depending on the external stimuli and internal metabolic state. In response to cancer-cell killing compounds, macrophages vigorously infiltrate the tumor and secrete tumorigenic factors, such as cathepsin (Shree et al., 2011; Alishekevitz et al., 2016), angiogenic factors (Alishekevitz et al., 2016) and chemokines (Welford et al., 2011), to promote tumor re-growth and metastasis. Cathepsin also secreted by MDSCs, further contribute to pro-tumoral recovery (Bruchard et al., 2013). Instead of supporting antigen presentation for a pro-tumoral response, cathepsin directly interacts with the NLRP3–IL-1β signaling pathway to induce IL-17 secretion by CD4+ T cells (Bruchard et al., 2013). This effect promotes angiogenesis and tumor progression. Chemotherapy also induces cancer cells to secrete more inflammatory IL-6 and prostaglandin E2, which drives monocyte differentiation toward tumor-promoting macrophages (Dijkgraaf et al., 2013). IL-6 can further coordinate with milk-fat globule-epidermal growth factor-VIII produced by macrophages to trigger STAT3 and Sonic Hedgehog pathways in cancer stem cells, thus amplifying their drug resistance (Jinushi et al., 2011). Oncolytic treatment also induces macrophages to produce large amounts of IL-10, which suppresses the anti-tumoral CD8+ T-cell response (Ruffell et al., 2014). Notably, most studies have described chemoprotective macrophages as having an M2-like phenotype, highlighting the importance of the macrophage phenotype in the outcome of cancer-cell mediated treatment.

### MDSCs in Chemotherapy

Myeloid-derived suppressor cells exhibit a clear immunosuppressive profile that restricts the efficacy of chemotherapy and often correlates with poor prognosis (Kawano et al., 2015; Takeuchi et al., 2015; Wesolowski et al., 2016). Cancer cells secrete more GM-CSF in response to chemotherapy, which induces monocyte differentiation into MDSCs. These newly differentiated MDSCs can suppress T-cell proliferation (Takeuchi et al., 2015). A significantly higher percentage of MDSCs after a completed round of chemotherapy is associated with a poor response and disease progression (Koinis et al., 2016). Limiting the MDSC population during adoptive immunotherapy using cytokine-induced killer cells has been shown to drastically improve survival in patients diagnosed with metastatic renal cancer, advanced pancreatic cancer and metastatic melanoma (Wang et al., 2016).

## Myeloid Cells Interfere With Immunotherapy

A greater understanding of onco-immunology will arm scientists with better knowledge as to how to mobilize the immune system in an effective fight against cancer. Immunotherapy has attracted vast attention as scientists began to demonstrate its efficacy in treating patients who are unresponsive to traditional anti-cancer methods. The landscape of cancer treatment has thus dramatically transformed with immunotherapy being used in many cases as a first-line treatment alongside conventional chemotherapy for highly refractory disease. In 2017, the number of immunotherapeutics on clinical trial reached a record high, with 467 ongoing studies registered just in the United States (Schmidt, 2017). Of these trials, T cells are the most commonly targeted immune cell type. Unfortunately, cancer cells readily secrete immunosuppressive compounds and express checkpoint ligands, thereby signaling to T cells to halt all killing activities. To circumvent this anti-T cell response, current therapeutic strategies aim to bolster anti-tumor T-cell activity with checkpoints inhibitors as a frontline cancer immunotherapy (Sharma et al., 2017).

### TAMs in Immunotherapy

Tumor-associated macrophages can impede the efficacy of checkpoint immunotherapy: *in vivo* imaging clearly showed that TAMs uptake anti-PD-1 monoclonal antibodies in tumor-bearing mice, thereby limiting the therapeutic antibody effect on PD1-expressing tumor-infiltrating CD8+ T cells (Arlauckas et al., 2017). In addition, evidence supports that reprogramming TAMs can alter efficacy of checkpoint drugs. For example, selective pharmacologic targeting of either gamma isoform of phosphoinositide 3-kinase (PI3Kγ) (De Henau et al., 2016; Kaneda et al., 2016), FcγR (Arlauckas et al., 2017), colony-stimulating factor 1 (CSF1R) (Zhu et al., 2014) or enzyme Arg1 (Steggerda et al., 2017) on myeloid cells suppresses their interaction with checkpoint drugs, thereby synergizing with T-cell targeted therapy to ensure effective targeting by checkpoint inhibitors. Killing TAMs seems an obvious solution to relieve immunosuppression in the tumor stroma. However, Zhu et al. (2014) demonstrated that treatment with CSF1R inhibitor, which effectively depletes TAMs, up-regulated PD-L1 expression on tumor cells and CTLA4 on T cells. Hence, depletion of TAMs essentially limits the anti-tumoral effects. Moreover, macrophage ablation could be detrimental to the host by increasing susceptibility to infection. Given that M2-like TAMs also function to control tumor growth under certain conditions (Grugan et al., 2012; Lin G.H.Y. et al., 2017), total macrophage ablation would not necessarily improve tumor outcomes. Instead, we propose taking advantage of their repolarization properties and enlisting these TAMs to fight against cancer.

### MDSCs in Immunotherapy

Similar to TAMs, MDSCs are obstructive to immunotherapy. An enhanced level of MDSCs was detected in patients with metastatic renal cancer, pancreatic cancer (Wang et al., 2016), colorectal cancer (Kanterman et al., 2014), metastatic pediatric sarcomas (Highfill et al., 2014) and non-small cell lung cancer (Delaunay et al., 2018). Combinational treatment with both immunotherapy and drugs targeting MDSC depletion drastically improved survival outcomes in cancer patients (Wang et al., 2016). Analysis attributed this favorable clinical outcome to reduced numbers of intratumoral MDSCs (Wang et al., 2016), which relieves T-cell suppression (Tongu et al., 2015). In a preclinical renal cell carcinoma mouse model, Rayman and colleagues observed that depleting MDSCs with TKI, sunitinib together with checkpoint blockade by anti-PD1 antibody co-treatment resulted in significantly more CD8+ T cells in the tumor (Rayman et al., 2015). This is accompanied by an increased production of pro-inflammatory IFNγ and granzyme B (Guan et al., 2017). More importantly, a high percentage of tumor-infiltrating CD8+ T cells expressed CD107a, which is involved in the cytotoxic killing of tumor target cells (Rayman et al., 2015). Other approaches such as monoclonal antibody therapy against CXCR2 (Highfill et al., 2014), CCL2 (Wang et al., 2018), or IL-18 (Guan et al., 2017) to inhibit MDSCs trafficking into tumors also successfully induces tumor regression upon anti-PD1 treatment. These studies suggest that resistance to immune-checkpoint blockade might be alleviated by therapeutic strategies that reprogram dominant myeloid responses.

## Targeting Warburg Metabolism in Myeloid Cells

As we have illustrated, myeloid cells, which are often immunosuppressive, have a diverse impact on cancer development, ranging from tumor progression to efficacy of cancer therapy. Therefore, it is imperative that we target these immune subsets during anti-cancer therapy. Rather than aiming for complete ablation and compromising our body to pathogens, re-polarizing myeloid cells to adopt an anti-tumoral profile is a promising avenue to explore. This way, macrophages can also be exploited to participate in and promote an anti-tumoral response. Most importantly, M2 macrophages can re-polarize into M1 macrophages in response to certain cytokines in the microenvironment (Davis et al., 2013). For example, blocking the CSF-1/CSF-1R axis or targeting the pattern recognition receptor MARCO causes a phenotypic shift from M2-like macrophages to M1-like macrophages resulting in increased tumor immunogenicity (Georgoudaki et al., 2016; Quaranta et al., 2018). In a glioblastoma multiforme mouse model, reduced expression of M2 markers accompanied with impaired pro-tumoral function was also observed (Pyonteck et al., 2013). In addition, sorafenib, a multikinase inhibitor, can reverse the immunosuppressive cytokine profile in tumor-conditioned macrophages, promoting them to elicit a more favorable anti-tumoral response (Edwards and Emens, 2010).

Administration of IFNγ, a stimulant of M1 polarization, achieved a favorable clinical outcome, with increased tumor cytotoxicity of TAMs in patients diagnosed with ovarian carcinoma (Allavena et al., 1990; Colombo et al., 1992). This clearly demonstrated the feasibility of immune remodeling in clinical settings. Given that the tumor microenvironment can condition myeloid cells toward a pro-tumoral phenotype, scientists can also take advantage of this plastic nature to reprogram myeloid cells toward an anti-tumoral phenotype that boost tumor control, hence improving treatment outcomes.

In general, pro-inflammatory M1 macrophages favor glycolysis whereas pro-wound healing M2 macrophages rely on oxidative metabolism (Galván-Peña and O’Neill, 2014). In the setting of the tumor microenvironment, pro-inflammatory M1-like macrophages target cancer cells and control tumor progression while pro-healing M2 macrophages promote tissue repair, assisting tumor growth (Helm et al., 2014b). However, TAMs do not simply display a distinct M1 or M2 profile. Rather, TAMs typically exhibit a mixed phenotype and several studies have demonstrated that glycolysis is essential to sustain these immunosuppressive cells (Helm et al., 2014b). Both our group and Zhao et al. (2017) reported that 2DG treatment can impede tumor growth *in vitro* and *in vivo* and transform TAMs into exhibiting an anti-tumoral phenotype (Penny et al., 2016). As cancer cells exhibit similar metabolic signature as pro-tumoral TAMs, currently available glycolytic drugs that target cancer cells might be relevant to reprogramming TAMs.

Glycolytic inhibitors, such as 3-bromopyruvate (3-BP) (Yun et al., 2009), MJE3 (Evans et al., 2005), 3-(3-pyridinyl)-1-(4-pyridinyl)-2-propen-1-one (3PO) (Clem et al., 2008), 3-dihydroxy-6-methyl-7-(phenylmethyl)-4-propylnaphthalene-1-carboxylic acid (FX11) (Le et al., 2010), and dichloroacetate (DCA) (Sutendra and Michelakis, 2013) target HK2, PFKFB3, PGAM1, LDHA and PDH, respectively in cancer cells to effectively suppress tumor growth. Genotoxic exposure induced by cisplatin blocks Glut1 and Glut3 expression on cancer cells, leading to a suppressed glycolytic rate and enhanced oxygen consumption (Zhou et al., 2002). Cisplatin is an appropriate drug to use since both glucose transporters are expressed on differentiated macrophages (Folco et al., 2011). HK2 is the first rate-limiting enzyme of the glycolytic pathway and silencing HK2 in hepatocellular carcinoma inhibits glucose flux to pyruvate and lactate, leading to cell death (DeWaal et al., 2018). Based on this concept, Ko et al. (2004) discovered 3-BP as an “incredible” anticancer agent that effectively eradicates all cancer growth in rodents within a short treatment regimen (Ko et al., 2004). TAMs harvested from tumor-bearing mice treated with 3-BP displayed enhanced tumoricidal activity and pro-inflammatory cytokine (IL-1, TNFα) production. However, TAM culture with 3-BP alone did not induce any changes in TAM cytotoxic function (Yadav et al., 2018) despite inhibiting the first step of glycolysis (Errea et al., 2016). Clearly, glycolytic drugs that are effective toward cancer cells might not necessarily be as effective toward TAMs. Further studies are now required to validate the effectiveness of these drugs on TAMs.

The ideal anti-cancer therapy would be to specifically eradicate tumor cells with minimal effect on non-neoplastic populations in the body. This feature is even more critical for glycolytic inhibitors since glycolysis is a metabolic pathway utilized by almost all mammalian cells. For example 3-BP, the most “promising” anti-cancer drug mentioned earlier, was associated with causing death in at least three cancer patients (DutchNews, 2016). Early *in vitro* and *in vivo* studies reported encouraging results showing the ability of 3-BP to inhibit tumor growth (Isayev et al., 2014; Liu et al., 2014; Gandham et al., 2015; Valenti et al., 2015; Zou et al., 2015), yet only two clinical trials, i.e., the studies on fibrolamellar hepatocellular carcinoma (Ko et al., 2012) and metastatic melanoma (El Sayed et al., 2014) were performed to further validate its effectiveness in human malignancies or examine the toxicity of the compound. *In vitro* studies using mouse/rat hepatocytes (Sobotka et al., 2016) and primary rat astrocytes (Ehrke et al., 2015) reported a dose-dependent toxicity of 3-BP (at doses ≥ 50 μM for hepatocytes and 100 μM for astrocytes) on non-neoplastic cells. In another study by Rodrigues and colleagues, they reported that 3-BP promotes a metabolic switch in embryonic stem cells resulting in the loss of pluripotency. However, the use of this drug alone is unlikely to drive these stem cells toward specific differentiation fates (Rodrigues et al., 2015). These *in vitro* data clearly showed that 3-BP can impact on healthy tissues, particularly those that rely heavily on glycolysis. As such, using the same glycolytic inhibitors to target myeloid cells will also encounter the exact same adverse effects. Therefore, delivery of drugs specifically to the tumor will be the game-changer for these glycolytic drugs to progress form bench to bedside.

## Concluding Remarks

Otto Warburg introduced the concept of Warburg metabolism, whereby tumor cells rely on glycolysis to support uncontrolled growth and proliferation (Warburg et al., 1927). We now know that tumor associated myeloid cells can also use this metabolic pathway. Under the influence of the tumor microenvironment, infiltrating myeloid cells undergo metabolic reprogramming to develop a new set of cellular functions. As these myeloid cells are responsive to environmental cues and are abundant within the tumor, exposure to metabolic drugs may prove to be an effective avenue to reshape these cells to kill cancer cells and alleviate T-cell suppression. Notably, the tumor stroma changes throughout cancer development, which also means that these dynamic myeloid cells must adopt unique metabolic states during cancer progression. There may be a therapeutic window when myeloid cells have nested within the tumor stroma but the fibrotic capsule has yet to form around the tumor. With growing interest in immunometabolism, continuous improvements in understanding the various metabolic pathways will provide new avenues to design formulated drugs that specifically target cancer cells and tumor associated myeloid cells in the future. In order to design strategies around this concept, improvements in metabolism-based therapeutics will be essential. Ultimately, immune-metabolism is only one arm of the cancer treatment strategy. Combinational treatment with targeted immunotherapy is recommended to successfully fight cancer.

## Author Contributions

JS, SG, and SW conceptualized the content and wrote the manuscript. JS and SG prepared the figures.

## Conflict of Interest Statement

The authors declare that the research was conducted in the absence of any commercial or financial relationships that could be construed as a potential conflict of interest.
